# Pulmonary embolism with floating right atrial thrombus successfully treated with streptokinase: a case report

**DOI:** 10.1186/s13104-016-2177-1

**Published:** 2016-07-28

**Authors:** Sahela Nasrin, Fathima Aaysha Cader, Md. Salahuddin, Tahera Nazrin, Masuma Jannat Shafi

**Affiliations:** Department of Cardiology, Ibrahim Cardiac Hospital & Research Institute (ICHRI), Dhaka, 1000 Bangladesh

**Keywords:** Massive pulmonary embolism, Right atrial thrombus, Streptokinase

## Abstract

**Background:**

Massive pulmonary embolism (PE) is associated with significant mortality, especially if compounded by haemodynamic instability, right ventricular (RV) dysfunction and right atrial (RA) thrombus. Thrombolysis can be lifesaving in patients with major embolism and cardiogenic shock, and accelerates the resolution of thrombus. Only three fibrinolytic agents—namely streptokinase, urokinase, and recombinant tissue plasminogen activator (alteplase) have been approved in the treatment of PE, with studies demonstrating similar safety profiles.

**Case presentation:**

We report the case of a 33-year-old Bangladeshi Bengali female with a history of recent ankle fracture and immobilization, who presented with massive PE, leading to cardiac arrest. Upon rapid resuscitation, urgent echocardiogram revealed RV dysfunction with floating RA thrombus, and she was successfully treated with 1.5 million IU of streptokinase over 2 h as per accelerated regimen recommended by the European Society of Cardiology guidelines, resulting in successful resolution of the right heart thrombus, and significant clinical improvement. Subsequent CT pulmonary angiogram confirmed the diagnosis of PE, and she was anticoagulated to a PT/INR of 2.0–3.0 for 3 months.

**Conclusions:**

Echocardiography is a suitable alternative for rapid diagnosis of acute massive PE associated with RA thrombus and cardiovascular collapse, especially when a delay to CT pulmonary angiogram may be anticipated, and in the setting of immediate cardio-pulmonary resuscitation. Thrombolysis is a rapid and life-saving therapeutic measure in such cases.

**Electronic supplementary material:**

The online version of this article (doi:10.1186/s13104-016-2177-1) contains supplementary material, which is available to authorized users.

## Background

Massive pulmonary embolism (PE) is frequently complicated with hypotension and shock, leading to mortality rates exceeding 50 % [1, 2]. Patients with right ventricular (RV) dysfunction are another subgroup with a guarded prognosis [3], as are those with right heart thrombus [4–6]. These patients in particular, benefit from more intensive therapy with thrombolytic agents in comparison to anticoagulant therapy alone, resulting in reduced mortality to less than 30 % [2, 6]. Thrombolytic therapy accelerates the resolution of PE, while reducing its recurrence and improving other parameters, such as pulmonary blood flow, lung perfusion, and RV dysfunction [4, 7]. Streptokinase, urokinase and recombinant tissue plasminogen activator (alteplase) are the thrombolytic agents approved for the treatment of PE, with alteplase being explicitly identified as the agent indicated for acute massive PE [7]. This case report aims to demonstrate the importance of prompt imaging and intervention, and the superior efficacy of thrombolysis in complicated massive PE.

## Case presentation

A 33-year-old normotensive, non-diabetic Bangladeshi Bengali female presented with sudden onset severe retrosternal chest pain and two episodes of syncope over 4 h. Chest pain was worse on deep inspiration and associated with shortness of breath, orthopnoea and palpitations for 2 days. She had an ankle fracture and was on a cast with plaster immobilization for the preceding month, and admitted to unilateral leg pain and swelling.

**Fig. 1:**
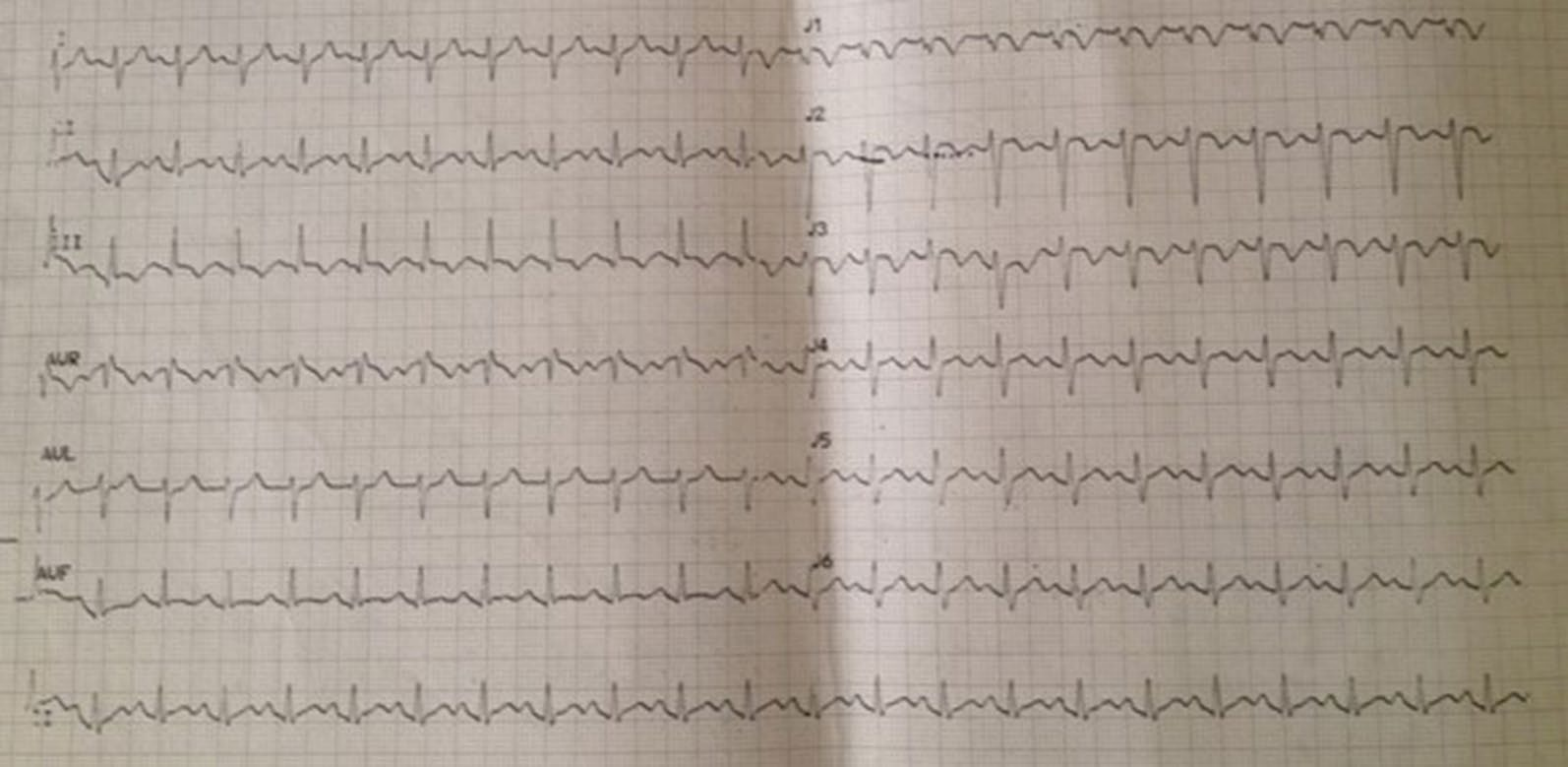
Electrocardiogram showing sinus tachycardia, right bundle branch block, “S-_1_Q-_3_T-_3_ pattern”

**Fig. 2:**
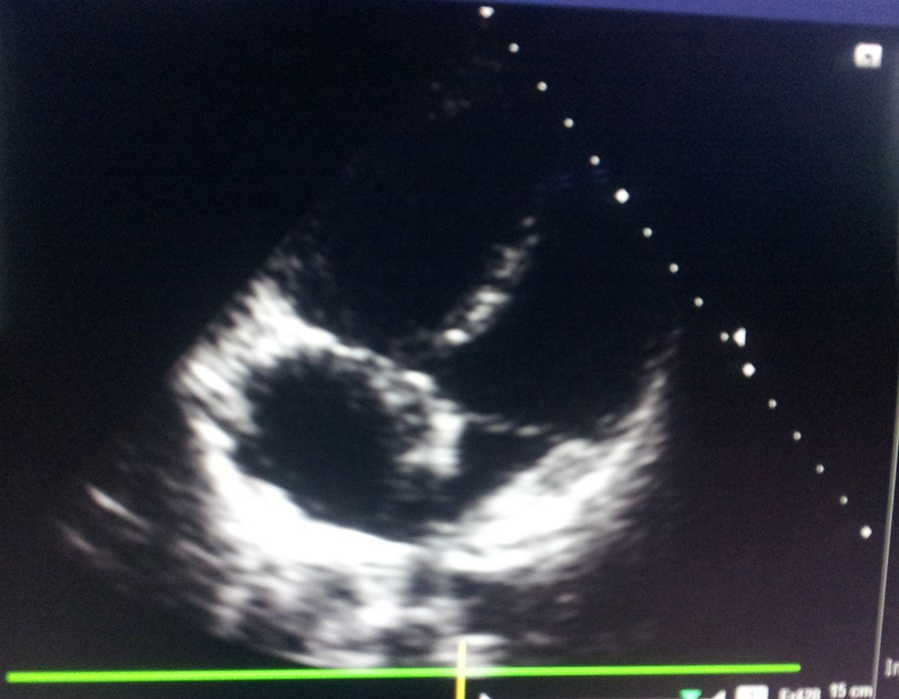
Trans-thoracic echocardiogram showing dilated right heart. Two-dimensional echocardiography in apical four chamber view showing dilated right atrium and right ventricle

**Fig. 3:**
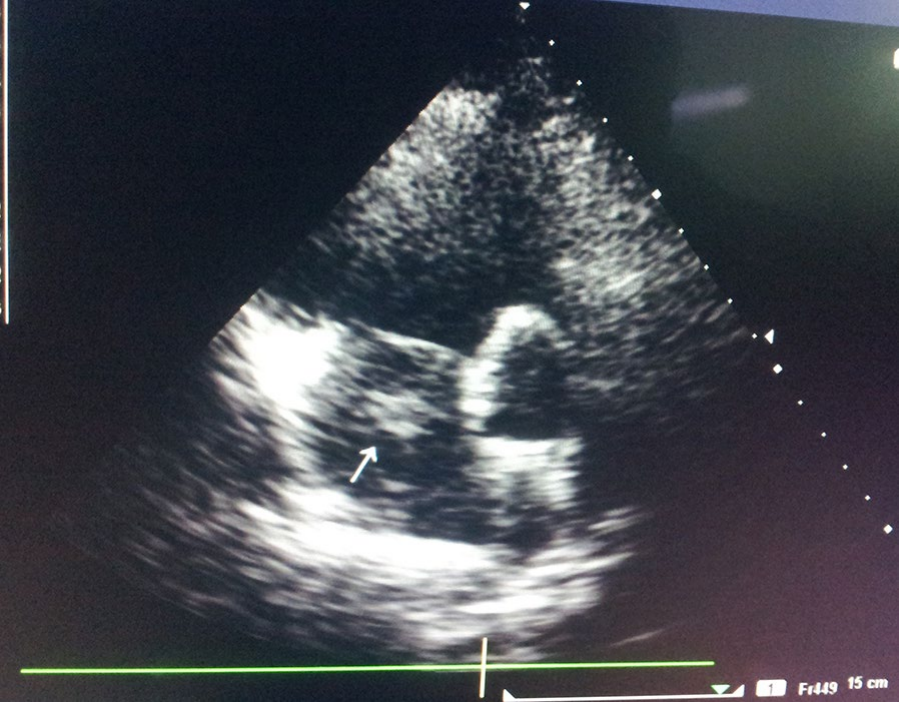
Trans-thoracic echocardiogram showing thrombus. Two-dimensional echocardiography in apical five chamber view showing thrombus (*white arrow*) in the dilated right atrium

**Fig. 4:**
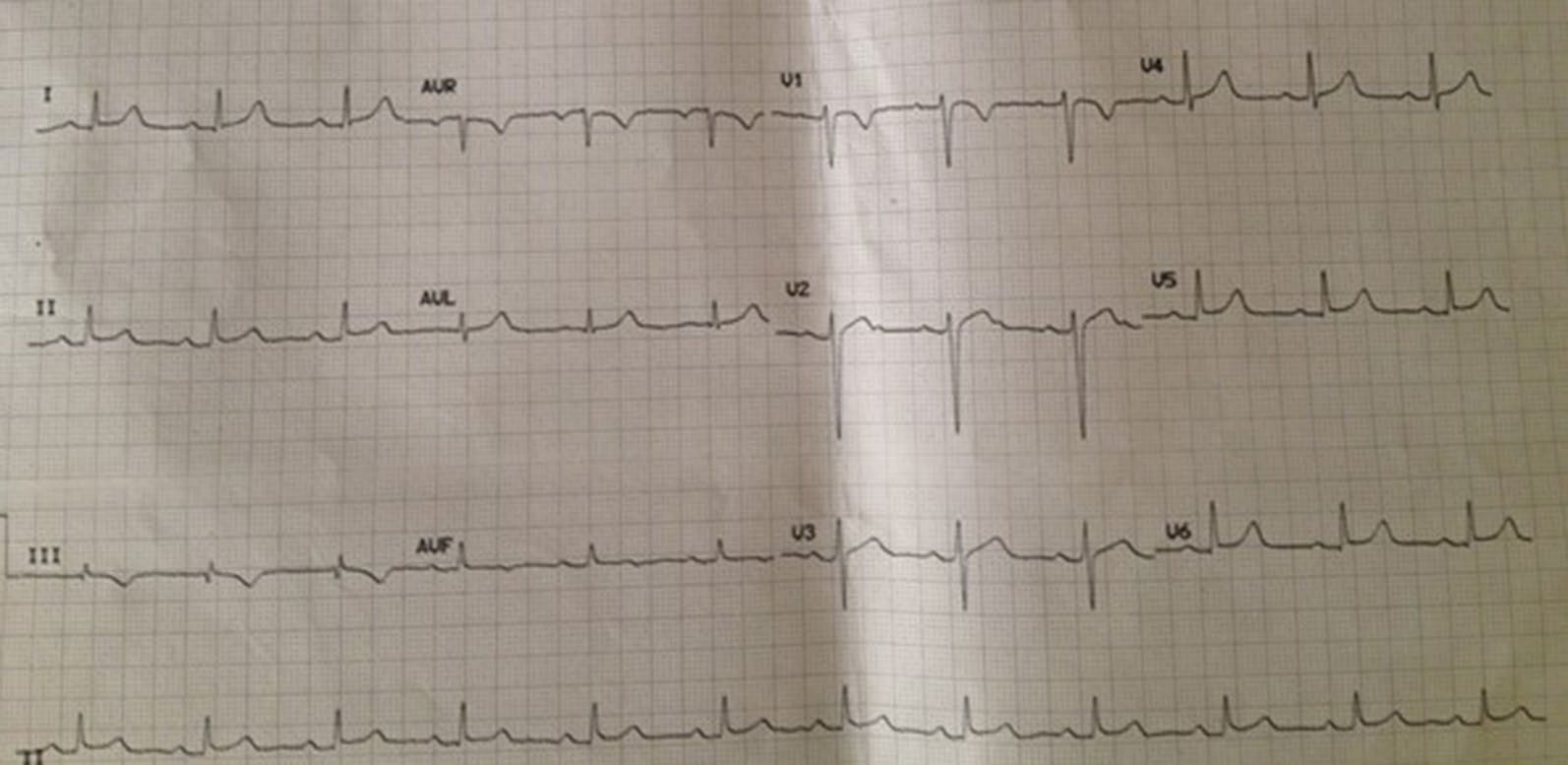
Electrocardiogram after thrombolysis, showing normal sinus rhythm, rate 72 beats/min

**Fig. 5:**
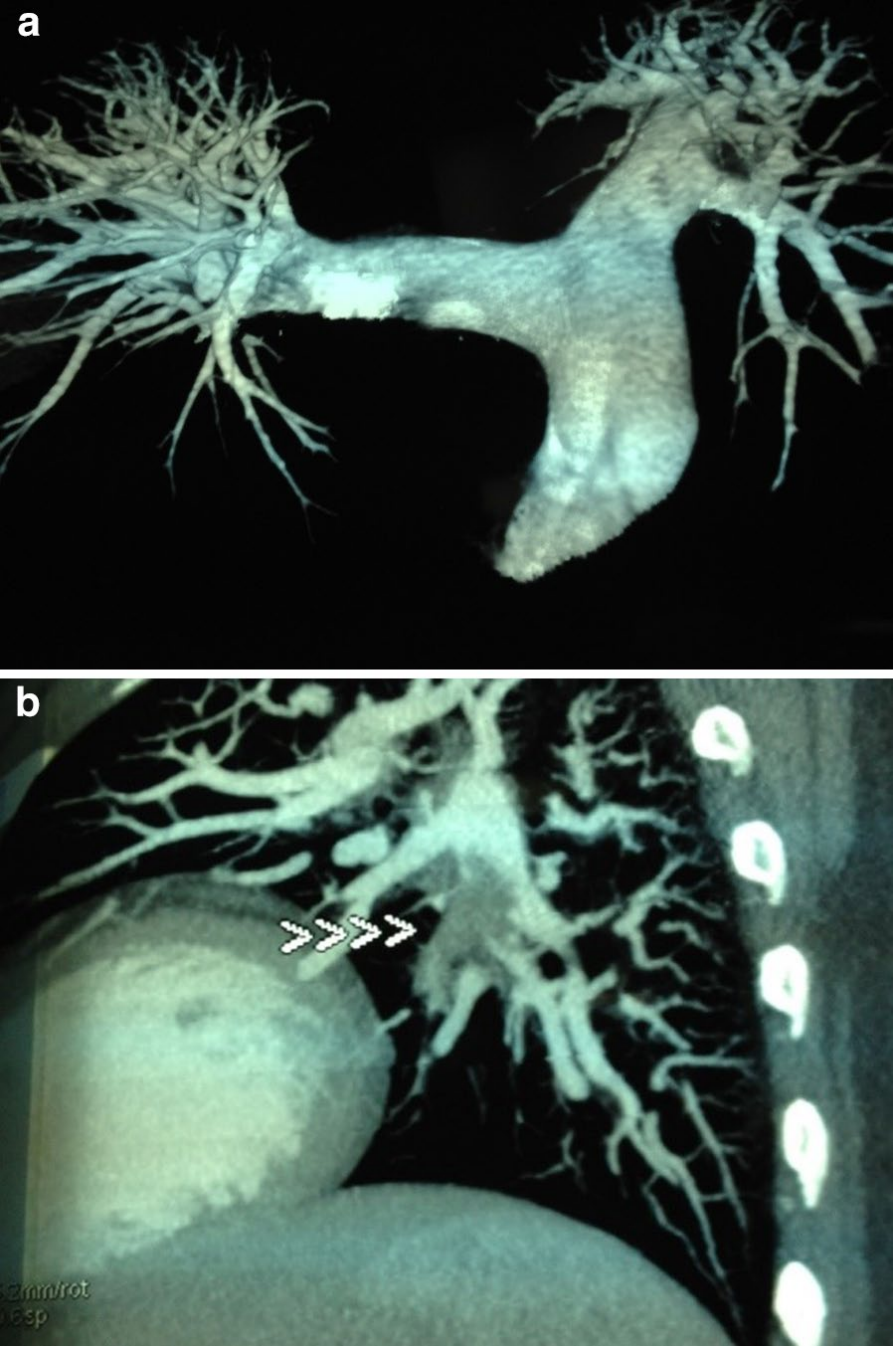
**a** CT pulmonary angiography showing thrombus. **b** CT pulmonary angiogram shows filling defects in descending branch of left pulmonary artery (*arrow heads* in **b**) extending up to segmental arteries, suggesting thrombus

On admission, she was cyanosed with gasping respiration; pulse and blood pressure were non-recordable. She developed asystole soon after, and reverted to sinus rhythm following 2 min of cardio pulmonary resuscitation (CPR). After resuscitation, heart rate was 136 beats/min and blood pressure was 80/55 mmHg. SpO2 was 90 %. Respiratory rate was 35 breaths/min. She was given high flow oxygen, intravenous (IV) normal saline and dopamine infusion for hypotension. Electrocardiogram (ECG) revealed sinus tachycardia (rate 136/min), right bundle branch block (RBBB) with S_1_Q_3_T_3_ pattern (Fig. 1). Bedside echocardiogram revealed dilated right atrium (RA) and RV (Fig. 2), floating thrombus in RA (Fig. 3; Additional file 1: Video S1); impaired RV systolic function [tricuspid annular planar systolic excursion (TAPSE) was 9 mm] with no evidence of RV hypertrophy; there was mild tricuspid regurgitation and pulmonary hypertension with pulmonary artery systolic pressure ~45 mmHg and normal left ventricular systolic function (ejection fraction 60 %). Immediate thrombolysis was done with IV streptokinase 1.5 million units over 2 h as per accelerated regimen of European Society of Cardiology (ESC) guidelines, resulting in a subsequently normal ECG (Fig. 4). Subsequent CT pulmonary angiogram done immediately revealed an approximately 2 cm filling defect in the descending branch of left pulmonary artery extending up to the lateral and posterior basal segmental arteries, suggesting thrombus (Fig. 5). d-dimer assay was positive. Troponin-I was 1.27 ng/mL (high risk—0.11–0.60). Complete blood count revealed neutrophilic leucocytosis. As she had no episodes of bleeding following thrombolysis, she was commenced on low molecular weight heparin (LMWH) at a dose of 1 mg/kg body weight for 5 days with simultaneous oral warfarin titrated to a therapeutic PT/INR of 2.0–3.0. Review echo done 2 days later revealed no thrombus or pulmonary hypertension, normal RA and RV. She was discharged on warfarin 5 mg daily and was asymptomatic with therapeutic PT/INR at 3 month follow up. She denied use of the oral contraceptive pill and was advised against its use owing to its potential as a risk factor. Hypercoagulability evaluation including Protein C and S levels, antinuclear antibody, anticardiolipin antibody and serum homocysteine levels were within normal range, thus eliminating other causes of hypercoagulability as possible aetiology of thrombosis.

## Discussion

This case of acute PE presented with cardiorespiratory arrest and is classified as massive PE as per American Heart Association (AHA) definitions [7]. The AHA defines massive PE as acute PE with sustained hypotension (systolic blood pressure <90 mmHg for at least 15 min or requiring inotropic support, not due to a cause other than PE), pulselessness, or persistent profound bradycardia (heart rate <40 bpm with signs or symptoms of shock) [7]. ESC guidelines also classify suspected acute PE as ‘high risk’ on the basis of presence of shock or hypotension [4].

Emergency multidetector CT should be performed in haemodynamically unstable suspected PE patients with shock or hypotension, because of its 97 % sensitivity for detecting emboli in the main pulmonary arteries [6, 7]. If unavailable, echocardiography should be performed without delay [4, 6, 7]. Echocardiographic markers of RV dysfunction, such as RV dilatation (without hypertrophy), paradoxical septal systolic motion, and pulmonary hypertension are independent predictive factors of poor outcome in acute PE [8]. Echocardiography can also detect right heart thrombi, a marker of worse prognosis, the prevalence of which is 4–18 % in the setting of an acute PE [9], and usually found in those more haemodynamically compromised [5, 6]. Free-floating right heart thrombi, are almost exclusively associated with PE [10, 11].

Elevated d-dimers and positive cardiac troponin T or I, both of which have a high negative predictive value, can be used for immediate risk stratification [4, 7, 12]. A normal d-dimer level renders acute PE or deep vein thrombosis (DVT) unlikely.

Scoring systems may be adopted for early risk stratification of patients, taking into account the clinical status and risk factors for venous thromboembolism (VTE) such as lower limb fractures, major trauma and surgery [7]. With a Well’s Score of 9, and a Revised Geneva Score of 11, our patient had high clinical probability of PE [13, 14]. According to 2014 ESC guidelines, she had high early mortality risk owing to shock, RV dysfunction on imaging and positive cardiac laboratory markers, thus warranting primary reperfusion [4]. There is no contraindication to fibrinolysis in cases of cardiac arrest owing to PE, however thrombolysis is discouraged in those with undifferentiated cardiac arrest [7]. Where patient transport for CT is unsafe, thrombolysis should be considered in case of unequivocal signs of RV overload on bedside echocardiography, and CT performed later [6].

There are three thrombolytics approved for the treatment of PE by the Food and Drug Administration (FDA): streptokinase, urokinase and alteplase, with alteplase being explicitly identified as the agent indicated for massive PE in 2010 [7]. There are no conclusive findings from studies comparing different thrombolytic regimens in acute PE, with most of them demonstrating similar safety profiles [15–17]. However, short infusion times (2 h or less) are recommended over prolonged infusion times, as they achieve more rapid thrombolysis and probably less bleeding [7, 18].

Thrombolytic treatment of acute PE restores pulmonary function more rapidly than anticoagulation with unfractionated heparin (UFH) alone [4]. Thrombolytic agents actively promote the hydrolysis of fibrin molecules, resulting in rapid resolution of thromboembolic obstruction. At 24 h, patients treated with adjunctive fibrinolysis manifest a 30–35 % reduction in total perfusion defect [7, 18]. This leads to a prompt reduction in pulmonary artery pressure and resistance, with a concomitant improvement in RV function, stabilization of respiratory and cardiovascular function, and prevention of PE recurrence [7]. Major contraindications include haemorrhagic or ischaemic stroke, recent major surgery or trauma or known bleeding risk [4, 17].

Thrombolysis has mortality benefit when compared either to anticoagulation or surgical thromboembolectomy, in cases of right heart thrombus [19]. A study by Rose et al. [19] revealed that the mortality rate associated with no therapy, anticoagulation therapy, surgical embolectomy, and thrombolysis was 100.0, 28.6, 23.8 and 11.3 %, respectively in patients with right heart thrombo-emboli. Thus, in PE in the presence of right heart thrombi, thrombolysis demonstrated an improved survival rate (p < 0.05) when compared with either to anticoagulation therapy or surgery.

Emergency surgical embolectomy or catheter embolectomy with fragmentation has been recommended for patients with massive PE or submassive PE with RV dysfunction, and contraindications to fibrinolysis or failed thrombolysis, provided appropriate expertise and resources are available [7, 20, 21]. Transfer to a centre in which these facilities are available should be considered for those patients with contraindication to thrombolysis or failed thrombolysis. The decision to proceed with either catheter-based or surgical embolectomy requires inter-disciplinary teamwork and operator expertise [7, 20]. Catheter-based embolectomy is generally reserved for cases in which neither thrombolysis nor surgical embolectomy is possible [1, 7], or in cases where thrombolysis has failed to improve haemodynamics in the acute setting. Operator expertise is essential [7]. Hybrid therapy that includes both catheter-based clot fragmentation and local thrombolysis is an emerging strategy [7].

Furthermore, in patients with acute PE, anticoagulation is recommended, with the objective of preventing both early death and recurrent symptomatic or fatal VTE [4]. This acute-phase parenteral anticoagulation may comprise of subcutaneous LMWH, IV or subcutaneous UFH or subcutaneous fondaparinux over the first 5–10 days, overlapping with oral vitamin K antagonist, warfarin [4, 7]. As our patient had already been thrombolysed and had no subsequent bleeding manifestations, she was commenced on LMWH.

LMWHs have many advantages over UFH. They have a greater bioavailability, can be administered by subcutaneous injections, and have a longer duration of anticoagulant effect. A fixed dose of LMWH can be used, and laboratory monitoring of activated partial thromboplastin time (aPTT) is not necessary [22].

Clinical trials comparing LMWH with UFH have shown that LMWH is comparable or superior to, and as safe as UFH [22]. Moreover, LMWH or fondaparinux are preferred over UFH for initial anticoagulation in PE, as they carry a lower risk of inducing major bleeding and heparin-induced thrombocytopenia [4]. Alternatively, UFH is recommended for patients in whom primary reperfusion is considered, as well as for those with serious renal impairment (creatinine clearance <30 mL/min), or severe obesity. Our patient received streptokinase immediately after resuscitation, following confirmation of RA thrombus by echocardiography, and suggestive ECG of acute PE. As such, this recommendation was not applicable in this situation, and LMWH was commenced and continued until therapeutic INR was achieved. As patients with acute PE are at risk for recurrent thromboembolism, they should be given long-term anticoagulation. The recommendation for PE secondary to a reversible risk factor is therapy with vitamin K antagonists for 3 months, titrated to a target INR of 2.0–3.0 [4, 6]. Novel oral anticoagulants (NOACs) i.e. dabigatran, rivaroxaban and apixaban are as effective and safe as warfarin for the treatment of VTE [4, 6, 7].

Follow up of patients is important, due to implications of long term anticoagulation and the possibility of chronic thromboembolic pulmonary hypertension after an acute PE, the incidence of which is up to 3.8 % 2 years after the acute event [23].

## Conclusions

Acute massive PE associated with RA thrombus, RV dysfunction and cardiovascular collapse is a cardiac emergency requiring prompt diagnosis and treatment, which can be life-saving. Echocardiography is a suitable alternative for rapid diagnosis of such cases, especially when a delay to CT pulmonary angiogram may be anticipated, and in the setting of immediate CPR. The effects of thrombolysis are extremely rewarding in cases of PE with cardiovascular collapse.

## Additional file

10.1186/s13104-016-2177-1 Trans-thoracic echocardiogram in apical four chamber view showing floating thrombus in right atrium.

## Abbreviations

PE: pulmonary embolism
RV: right ventricular
CPR: cardio pulmonary resuscitation
ECG: electrocardiogram
RBBB: right bundle branch block
RA: right atrial
TAPSE: tricuspid annular planar systolic excursion
ESC: European Society of Cardiology
IV: intravenous
LMWH: low molecular weight heparin
UFH: unfractionated heparin
PT/INR: prothrombin time/international normalisation ratio
AHA: American Heart Association
DVT: deep vein thrombosis
VTE: venous thromboembolism
FDA: Food and Drug Administration
aPTT: activated partial thromboplastin time
NOAC: novel oral anticoagulant
